# Individual differences in social play behaviour predict alcohol intake and control over alcohol seeking in rats

**DOI:** 10.1007/s00213-021-05929-1

**Published:** 2021-08-02

**Authors:** Heidi M. B. Lesscher, E. J. Marijke Achterberg, Stephen M. Siviy, Louk J. M. J. Vanderschuren

**Affiliations:** 1 Department of Population Health Sciences, Unit Animals in Science and Society, Faculty of Veterinary Medicine, Utrecht University, Yalelaan 2, 3584 CM Utrecht, The Netherlands; 2 Department of Psychology, Gettysburg College, Gettysburg, PA 17325 USA

**Keywords:** Social play, Alcohol, Addiction, AUD, Loss of control, Individual differences, Rats

## Abstract

**Rationale:**

Social play behaviour is a rewarding social activity displayed by young mammals, thought to be important for the development of brain and behaviour. Indeed, disruptions of social play behaviour in rodents have been associated with cognitive deficits and augmented sensitivity to self-administration of substances of abuse, including alcohol, later in life. However, the relation between social development and loss of control over substance use, a key characteristic of substance use disorders including alcohol use disorder (AUD), has not been investigated. Moreover, it remains unknown how inherent differences in playfulness relate to differences in the sensitivity to substance use and AUD.

**Objective:**

The objective of this study is to determine how individual differences in juvenile social play behaviour predict alcohol intake and loss of control over alcohol seeking.

**Methods:**

Juvenile male Lister hooded rats were characterized for their tendency to engage in social play behaviour. Subsequently, alcohol consumption and conditioned suppression of alcohol seeking were assessed in the tertiles of rats that showed the most and least social play.

**Results:**

The rats that engaged most in social play behaviour consumed more alcohol than their less playful counterparts. However, whereas the most playful rats showed intact conditioned suppression of alcohol seeking, the least playful rats showed no such suppression.

**Conclusion:**

Individual levels of playfulness predict the sensitivity to alcohol-directed behaviour. Highly playful rats are more prone to alcohol intake, yet show greater control over alcohol seeking. These findings increase our understanding of the relationship between social development and vulnerability to AUD.

**Supplementary Information:**

The online version contains supplementary material available at 10.1007/s00213-021-05929-1.

## Introduction

Social play behaviour is the most prominent form of social behaviour displayed by young mammals (Panksepp et al. 1984; Pellis and Pellis 2009; Vanderschuren et al. 1997). Social play behaviour is highly rewarding (Achterberg et al. 2016a; Achterberg et al. 2016b; Calcagnetti and Schechter 1992; Humphreys and Einon 1981; Mason et al. 1962; for review see: Trezza et al. 2011) and thought to be important for the development of brain and behaviour (Graham and Burghardt 2010; Pellis and Pellis 2009; Piaget 1962; Spinka et al. 2001; Vanderschuren and Trezza 2014). Indeed, disruptions of social play behaviour in rodents have been associated with cognitive deficits and aberrant social behaviour in adulthood (Baarendse et al. 2013; Einon et al. 1978; Hol et al. 1999; van den Berg et al. 1999; Von Frijtag et al. 2002). Social play behaviour is modulated through neural systems that also mediate the rewarding effects of natural rewards like food or sex, as well as substances of abuse (Siviy and Panksepp 2011; Trezza et al. 2010; Vanderschuren et al. 2016). Substances of abuse alter the expression of play behaviour (for review see Trezza et al. 2014; Vanderschuren et al. 2016). For example, treatment with low doses of alcohol enhances social play behaviour in rats (Trezza et al. 2009; Varlinskaya et al. 2001, 2010; Varlinskaya and Spear 2002). Moreover, play deprivation in rats has been shown to result in enhanced sensitivity for cocaine self-administration (Baarendse et al. 2014) and for cocaine-, amphetamine- and alcohol-induced conditioned place preference (Walker et al. 2020; Whitaker et al. 2013). Also, play deprivation augmented alcohol consumption in adulthood (Lesscher et al. 2015). Together, these studies suggest a critical role of social play in the development of the brain mechanisms underlying positive emotions and cognitive control, with consequences for, amongst others, reward sensitivity and addictive behaviour (Trezza et al. 2014).

In recent studies, we demonstrated a high degree of individual variation in alcohol consumption in Lister hooded rats, which was paralleled by variation in the loss of control over alcohol seeking (Spoelder et al. 2015, 2017), which is a key characteristic of substance use disorders, including alcohol use disorder (AUD) (American Psychiatric Association 2013). In the present study, we sought to investigate the importance of social play behaviour for the sensitivity to develop AUD-like behaviour in rats. The aim of this study was therefore to determine the relation between individual variation in social play behaviour, alcohol consumption and loss of control over alcohol seeking. To assess that, we characterized juvenile Lister hooded rats for their tendency to engage in social play behaviour and subsequently characterized these animals for their degree of alcohol consumption (both during adolescence and adulthood) and control over alcohol seeking in adulthood. Considering that play deprivation enhances the risk for addictive behaviour (Baarendse et al. 2014; Lesscher et al. 2015; Whitaker et al. 2013), we hypothesized that juvenile animals that played most would be less prone to consume alcohol and lose control over alcohol seeking.

## Methods

### Animals

Male Lister hooded rats (Charles River, Sulzfeld, Germany) arrived at postnatal day 21 in the facility and were initially group housed (4 per cage) under controlled conditions, with a reversed 12-h light/dark cycle (lights off 7.00 AM) and ad libitum access to water and chow. A paper towel and a Perspex shelter were available as home cage enrichment. The rats were acclimatized to the facility for 1 week upon arrival and were weighed and handled at least once per week. After completion of the play experiments, from postnatal day 42, the animals were individually housed for the remainder of the study. For logistical reasons, the entire study was performed in two batches (48 rats each). All experimental procedures were approved by the Central Authority for Scientific Procedures on Animals and were conducted in accordance with Dutch (Wet op de Dierproeven, 2014) and European legislation (Guideline 86/609/EEC; Directive 2010/63/EU).

### Experimental design

**Fig. 1:**
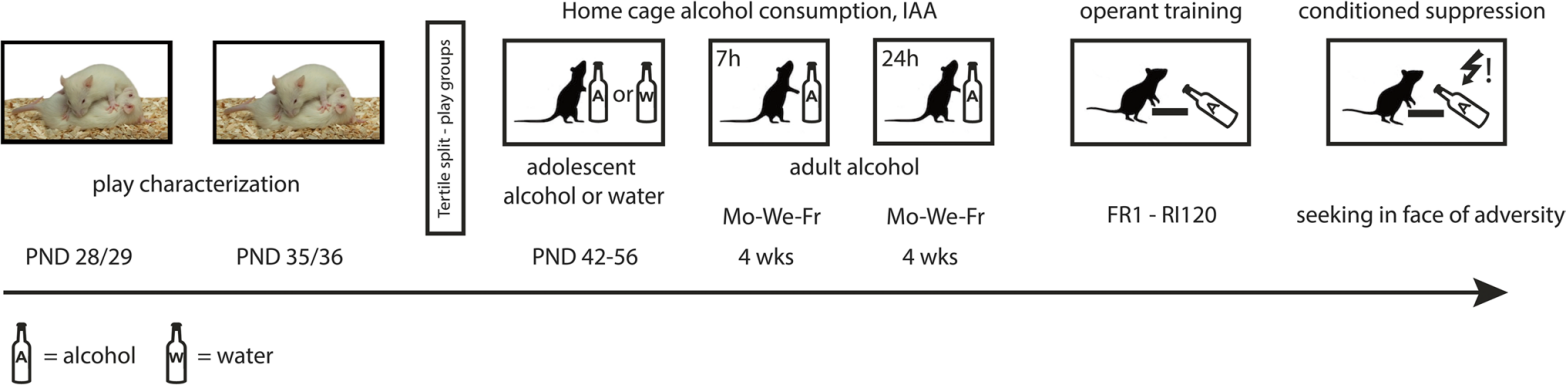
Experimental timeline

In this study, we compared rats that differ in their tendency to engage in social play behaviour for their degree of alcohol consumption and their control over alcohol seeking (see Fig. 1). For this purpose, the rats were first characterized for their tendency to engage in social play behaviour on postnatal day 28/29 and 35/36. Subsequently, the levels of voluntary alcohol consumption for these same animals were determined by exposing them to alcohol, either during adolescence (postnatal day 42–56) and adulthood or during adulthood only. Thereafter, the rats were trained to self-administer alcohol and ultimately control over alcohol seeking was determined using a conditioned suppression test (Spoelder et al. 2017; Vanderschuren and Everitt 2004).

### Social play behaviour

Rats were tested two times for their tendency to engage in social play behaviour, on postnatal day (PND) 28/29 and 35/36. Each animal was habituated twice to a type III cage (Tecniplast, Varese, Italy) for 10 min on PND 23 and 27. On the test day, each rat was isolated for 2.5 h prior to testing, in a Eurostandard type III cage in a room different from the housing room, to increase their motivation for social play behaviour to half maximal levels and prevent floor and ceiling effects (Achterberg et al. 2018; Niesink and Van Ree 1989; Vanderschuren et al. 1995, 2008). On both test days, the rats were paired such that they could all play with an unfamiliar partner of approximately the same weight (maximal difference of 10 g). Testing consisted of placing a pair of rats in a play setup (Plexiglas cage, 40 × 40 × 60 cm) for 15 min. Each play session was recorded using an infrared video camera and a DVD recorder. The behaviour of both rats was assessed live by a trained observer from a television monitor in an adjacent room using Observer 5.1 software (Noldus Information Technology). The following behavioural parameters were scored: pouncing, pinning, being pounced, being pinned, social exploration and non-social exploration. A play bout in rats commences with one rat ‘pouncing’ on another animal, by attempting to nose or rub the nape of its neck. This is considered solicitation for play, to which the pounced-upon partner can respond in several ways. It can move away and the soliciting rat can subsequently respond by chasing its play partner, trying to initiate another play bout. Alternatively, the animal may fully rotate to its dorsal surface, resulting in ‘pinning’, which is defined as one animal lying on the floor with its dorsal surface and the other animal standing over it. From this position, a new play bout can be initiated (Panksepp and Beatty 1980; Pellis and Pellis 1987; Poole and Fish 2009; Trezza et al. 2010).

To divide the rats into rats with a consistent low, medium or high tendency to engage in social play behaviour, ranking scores were assigned based on the active pounces and pins on each test day, separately for the two batches. Subsequently, the sum of the ranking scores for active pounces and pins was calculated. Based on these total rank scores, the population was subdivided into low, medium and high playing rats (LP, MP and HP, respectively) using a tertile split. Rats that deviated more than 35% in their number of active pounces from one test day to the other were excluded from further analyses because these animals could not with certainty be classified as LP, MP or HP.

### Voluntary home-cage alcohol consumption

A total of 96 rats were characterized for their tendency to engage in social play behaviour. Half of the rats were subsequently exposed to alcohol in 7-h consumption sessions during adolescence (from PND 42 to PND 56); the other half only consumed water but were otherwise treated the same. The rats were given access to 20% (v/v) alcohol (Klinipath, The Netherlands) and water in a two-bottle choice setup in the home cage with intermittent alcohol access (IAA) for 3 days a week (Monday-Wednesday-Friday). After this period of adolescent alcohol consumption, the rats were all left undisturbed until adulthood. From PND 75 onwards, all rats were allowed to drink alcohol in a two-bottle choice setup, as above, for two consecutive months. During the first month of adulthood alcohol consumption, alcohol was presented for 7 h/day (9.00 AM until 16.00 PM) and access to alcohol was extended to 24 h/day thereafter (Spoelder et al. 2015). Alcohol intake (g/kg) was calculated per rat per session and subsequently averaged per week.

### Alcohol self-administration

#### Operant training

After 2 months of voluntary home-cage alcohol consumption, the rats were trained to lever press for alcohol in operant conditioning chambers (Med-Associates, USA) 3 days/week (Monday-Wednesday-Friday) as previously described (Spoelder et al. 2017). The position of the active and inactive levers was counterbalanced between rats. Upon meeting the response requirement, the dipper cup containing alcohol (0.1 ml, 20% v/v) was raised, the cue light was illuminated above the active lever and the house light was switched off. Ten seconds after a head entry into the magazine, access to alcohol was terminated, the cue light was turned off and 5 s later a new trial started. The rats were initially trained in 30-min sessions under a fixed ratio 1 (FR1) schedule of reinforcement, and subsequently under random interval (RI) schedules of reinforcement (3 × RI 5 s, 3 × RI 15 s, 2–3 × RI 30 s and 2–3 × RI 60 s), in line with previous conditioned suppression studies (Limpens et al. 2014a, 2014b; Spoelder et al. 2017; Vanderschuren and Everitt 2004). Finally, the rats were trained in five 60-min RI 120-s sessions. Random interval schedules of reinforcement allow for the dissociation of seeking and taking behaviour (Olmstead et al. 2000). Moreover, random interval schedules produce steady seeking response rates with infrequent presentation of the actual reward, and therefore allow for tests in extinction, the conditioned suppression test in this case, without interference of the pharmacological effect of the substance itself. Stable responding was defined as < 25% variation in active responses during the RI in the first 15 min of the last three RI 120-s sessions. Experimental events and data recording were controlled using MED-PC software.

#### Conditioned suppression of alcohol seeking

The conditioned suppression test was performed according to previously described procedures (Limpens et al. 2014a, 2014b; Spoelder et al. 2017; Vanderschuren and Everitt 2004). To avoid bias in the conditioned suppression of alcohol seeking due to baseline differences in alcohol intake, the rats were assigned to groups that either underwent fear conditioning, with conditioned stimulus (CS)-footshock pairings (CS +), or underwent control conditioning (CS −), as described previously, taking into account the rats’ average alcohol intake per week and their mean seeking responses per minute during the first 15 min of the last three RI 120-s sessions. The data was ultimately analysed by play categories, as described in the previous section. In total, 9 LP and 13 HP underwent control conditioning (CS −) and 7 LP and 16 HP were fear-conditioned.

Acquisition of the CS-shock association was established in conditioning chambers that were physically different from operant self-administration (SA) chambers as described previously (Spoelder et al. 2017). The rats were habituated to the conditioning chambers in three 30-min sessions prior to conditioning. A CS-shock conditioning session started with a 5-min period in which only the house light was illuminated, followed by two periods of 10 min during which an 85-dB, 2900-Hz tone (separated by a 10-min inter-trial-interval) was constantly presented. During the 10-min tone presentations, 10 unpredictable, scrambled footshocks (0.4 mA, 1-s duration) were delivered to a total of 20 shocks for each CS + rat. The conditioning session was completed after a final 5-min period without tone presentation. Rats in the CS − control group underwent the same procedure, but without footshocks.

After two additional RI120 sessions, conditioned suppression of alcohol-seeking behaviour was assessed in the SA chambers. The house light was illuminated and 2 min after the start of the session, the levers were extended for the remaining 12 min of the test. Alcohol seeking during the conditioned suppression test was examined in extinction, i.e. responding on the levers was recorded, but had no programmed consequences. The cup containing 20% alcohol (v/v) was present underneath the liquid dipper but the rats did not have access to the solution. Two-minute intervals in which the tone CS was presented (CS-ON interval) were alternated with two-minute intervals where the tone CS was absent (CS-OFF interval). Active lever presses and latency to the first lever press were recorded and compared for CS − and CS + subgroups as a measure for control over alcohol seeking.

#### Conditioned freezing

One week after completion of the conditioned suppression test, the rats underwent fear conditioning (CS +) or control conditioning (CS −); the rats were assigned to the same group as before. The fear conditioning procedures were similar to those described in the previous section. Twenty-four hours after this fear conditioning session, the rats were placed in the conditioning chamber: 2 min without the CS + tone and subsequently 2 min with the CS + tone. The frequency and duration of freezing behaviour, defined as the absence of any movement other than breathing (Blanchard and Blanchard 1969; Bouton and Bolles 1980; LeDoux et al. 1984), was scored from DVD-taped behaviour using Observer software (Noldus, Wageningen, The Netherlands) by an observer blind to treatment.

### Statistical analyses

The social play data were analysed by multivariate analyses, with play subgroup as the between-subjects variable. For further analyses, to compare the most extreme groups in terms of social play behaviour, only the lowest and highest playing animals (i.e. LP and HP) were considered. The alcohol consumption data was analysed by two-way repeated measures ANOVA with time (weeks) as the within-subjects factor and adolescent alcohol/water or alcohol and playgroup (LP or HP) as the between-subjects factors.

For the analysis of the conditioned suppression data, the data for the adolescent water- and alcohol-consuming rats were combined because (1) adolescent alcohol exposure did not affect conditioned suppression and (2) the group sizes were very small after exclusion of those rats that showed inconsistent play behaviour across the two play sessions, in particular for the low players (*N* = 3–6). The conditioned suppression data were initially analysed using a four-way repeated measures ANOVA with playgroup and CS group (CS − and CS +) as the between-subjects factors and tone (no-tone vs. tone) and interval (CS-ON and CS-OFF, 2-min periods) as the within-subjects variables. Subsequently, the conditioned suppression data were also analysed separately per playgroup, using three-way repeated measures ANOVA with CS group (CS − and CS +) as the between-subjects factor and tone (no-tone vs. tone) and interval (CS-ON and CS-OFF, 2-min periods) as the within-subjects variables. A significant difference in the number of lever presses and/or latency to respond for alcohol between the CS − and CS + groups is indicative of control over alcohol seeking, while a non-significant difference between CS − and CS + animals indicates loss of control over alcohol seeking on a group level. The conditioned fear data were analysed by three-way repeated measures ANOVA with CS group (CS − and CS +) and play group as between-subjects factors and interval (tone-OFF vs. tone-ON) as a within-subjects factor. Mauchly’s test of sphericity was used to test if variances of the differences between treatment levels were equal. If the assumption of sphericity was violated, degrees of freedom were corrected using Huynh–Feldt estimates of sphericity to more conservative values; corrected degrees of freedom are presented rounded to the nearest integer. When appropriate, post hoc analyses were conducted using Student’s *t*-tests or pairwise comparisons. Each parameter was tested for normality with a Kolmogorov–Smirnov test. In case the behavioural parameters were not normally distributed, data was square root transformed (active responses conditioned suppression test and conditioned freezing) or log transformed (latency data conditioned suppression test) prior to statistical analyses, which resulted in normal distribution of the data. Statistical analyses were conducted using IBM SPSS Statistics for Windows, version 22.0 (IBM Corp., Armonk, NY, USA). The threshold for statistical significance was set at *p* < 0.05. All data are presented as mean + SEM.

## Results

### Play behaviour

**Fig. 2:**
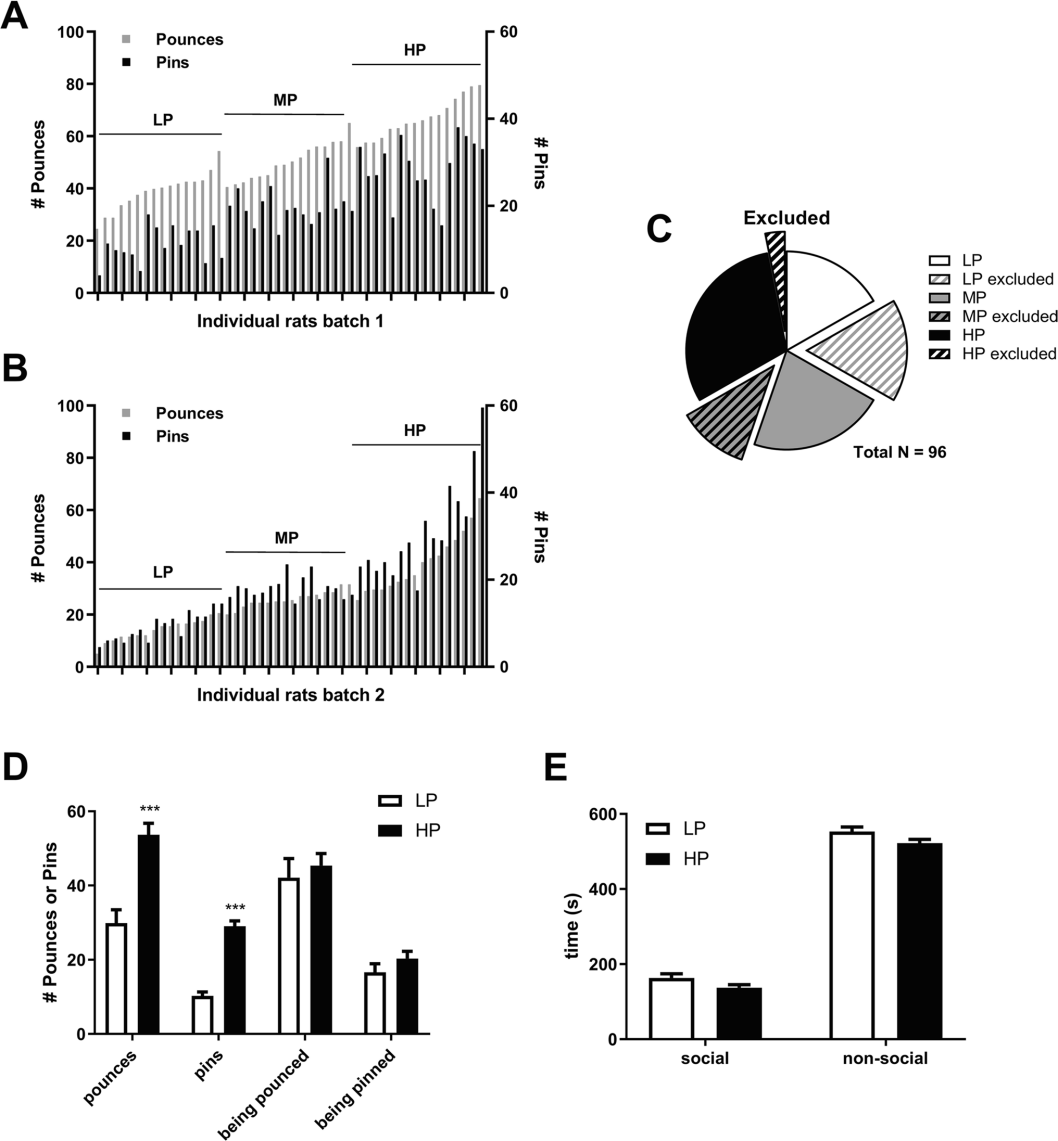
Social play behaviour for all individual rats. **A** and **B** The number of pounces (grey) and pins (in black) for all individual animals in batch 1 and 2, respectively (*N* = 48 per batch); LP, low players; MP, medium players; HP, high players. **C** The distribution of the rats that were excluded because they could not with certainty be classified as LP, MP or HP. **D** The average number of pounces and pins performed and the number of occasions the animals were being pounced or pinned are shown for the groups of selected LP (*N* = 16) and HP (*N* = 29). **E** The average time spent on social or non-social exploration for the selected groups of LP (*N* = 16) and HP (*N* = 29). Data are shown as average + SEM by play subgroup. ***Significant from low playing rats (*p* < 0.001)

A total of 96 rats were characterized for their tendency to engage in social play behaviour. Figures 2A and B summarize the number of pounces and pins made by the individual rats for batch 1 and 2, respectively. Thirty rats were excluded from further analyses because of high variation in play behaviour from one test day to the other, defined by more than 35% deviation between the two test days in the number of active pounces. These rats could therefore not with certainty be classified as LP, MP or HP (see Fig. 2C for the distribution across the subgroups). HP made more pounces (F_(1,44)playgroup_ = 22.6, *p* < 0.001) and pins (F_(1,44)playgroup_ = 79.0, *p* < 0.001) compared to LP (Fig. 2D). However, there were no differences between LP and HP in the amount of pounces or pins they received (F_(1,44)playgroup_ = 0.3, N.S. and F_(1,44)playgroup_ = 1.3, N.S., respectively) (Fig. 2D). Moreover, the LP and HP spent equal amounts of time on social (F_(1,44)playgroup_ = 3.8, N.S.) and non-social exploration (F_(1,44)playgroup_ = 3.7, N.S.) (Fig. 2E).

### Alcohol intake

**Fig. 3:**
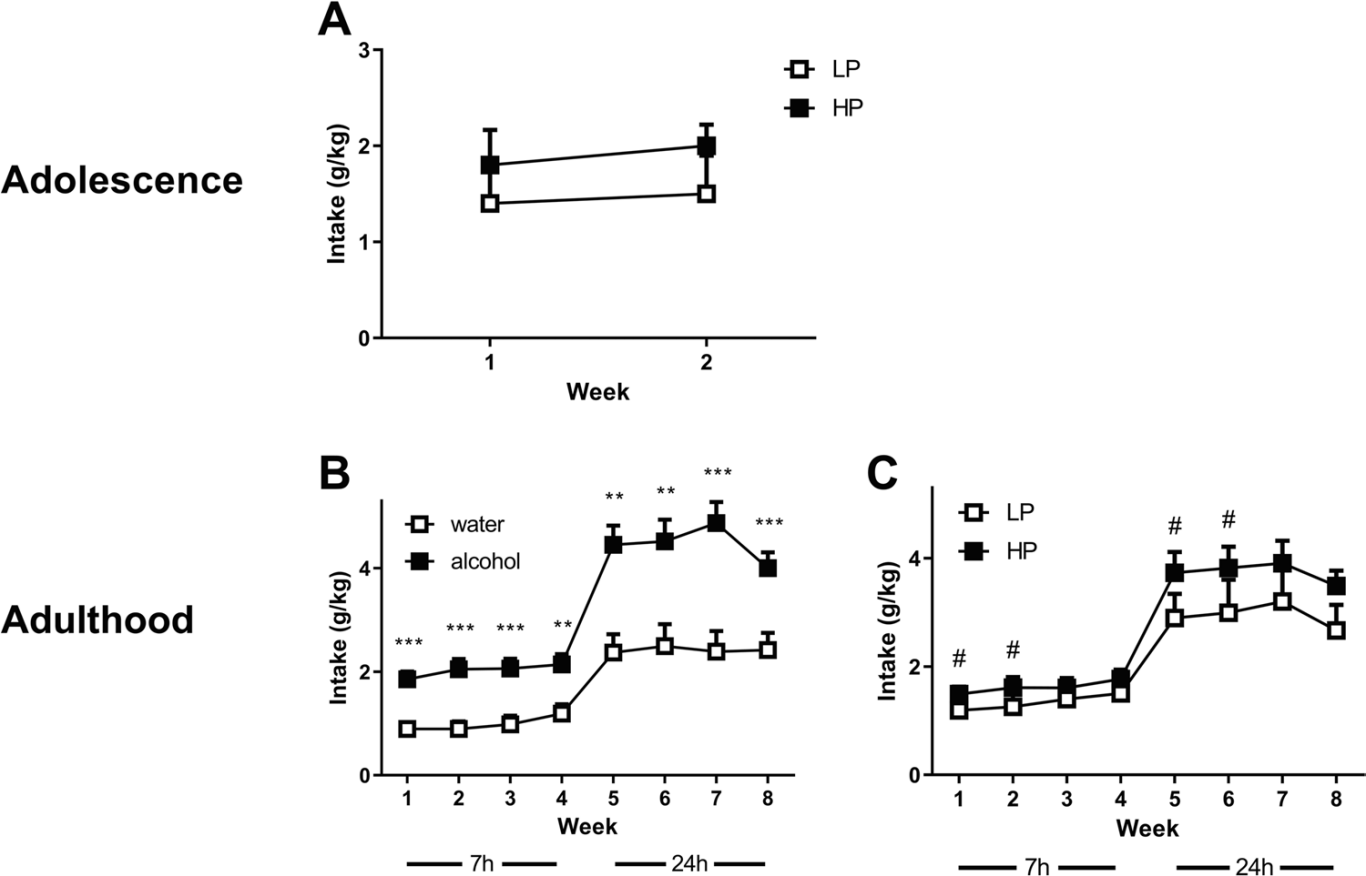
Alcohol intake for the selected groups of low and high playing rats that were allowed to drink either alcohol or water during adolescence. **A** The alcohol intake during adolescence is shown (LP *N* = 9, HP *N* = 14). **B** The levels of alcohol intake (g/kg) in adulthood, in 7-h (weeks 1–4) and 24-h (weeks 5–8) sessions, by water (*N* = 22) or alcohol (*N* = 23) pre-exposure. **C** The levels of alcohol intake (g/kg) for the same rats, grouped by low (LP, *N* = 16) or high (HP, *N* = 29) tendency to engage in social play behaviour. Shown are the average + SEM levels of weekly alcohol intake per group. Post hoc pairwise comparisons: ** or *** significant difference between adolescent water and adolescent alcohol (*p* < 0.01 or *p* < 0.001, respectively); # significant difference between low and high playing rats (*p* < 0.05)

The LP and HP rats did not differ in their level of alcohol consumption during adolescence (Fig. 3A; F_(1,21)time × playgroup_ = 0.07, N.S. and F_(1,21)playgroup_ = 1.1, N.S.). In adulthood, the rats that had consumed alcohol during adolescence showed higher levels of alcohol intake compared to the animals that had not consumed alcohol during adolescence (F_(1,40)AdolAlc_ = 18.5, *p* < 0.001 and F_(7,280)time × AdolAlc_ = 6.4, *p* < 0.01, Fig. 3B). Moreover, the HP overall consumed more alcohol than the LP in adulthood (F_(1,40)playgroup_ = 5.4, *p* < 0.05, Fig. 3C). In line with these findings, the rats that had consumed alcohol during adolescence showed a higher preference for alcohol compared to rats that were not previously exposed to alcohol (F_(1,40)AdolAlc_ = 18.0, *p* < 0001), irrespective of the week of alcohol consumption in adulthood (F_(5.7,229)time × AdolAlc_ = 1.7, N.S.) (Supplementary Fig. 1A). However, the low and high playing rats did not differ in their preference for alcohol over water (F_(1,40)playgroup_ = 3.1, N.S.; F_(5.7,229)time × playgroup_ = 0.8, N.S.) (Supplementary Fig. 1B).

### Conditioned suppression of alcohol seeking

**Fig. 4:**
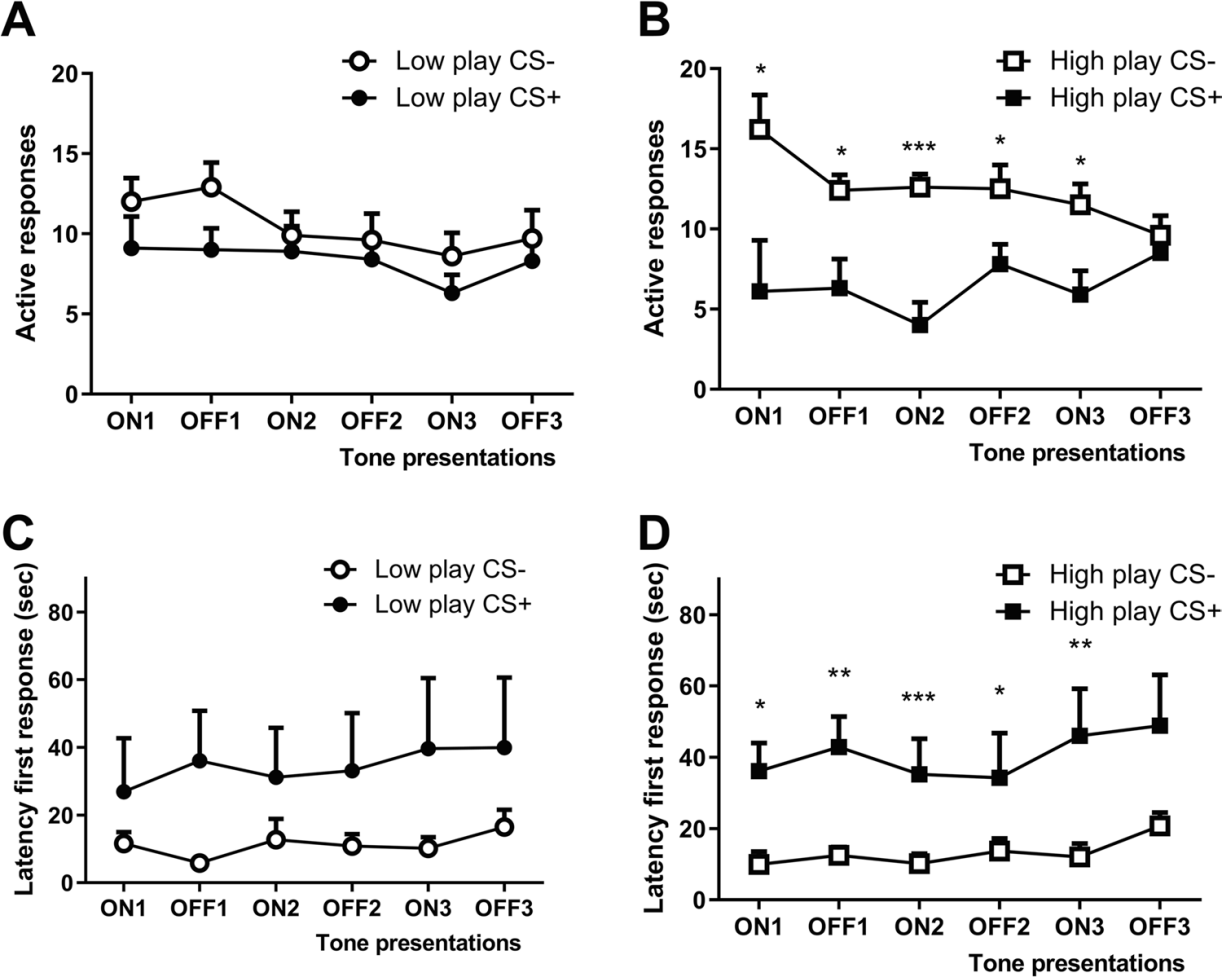
Conditioned suppression of alcohol seeking in low and high playing rats after 8 weeks of intermittent alcohol consumption. The number of active responses during consecutive CS-ON and CS-OFF intervals is shown for low players (**A** LP, CS − *N* = 9; CS + *N* = 7) and high players (**B** LP, CS − *N* = 13; CS + *N* = 16) that were control conditioned (CS −) or conditioned to associate a tone with footshocks (CS +). The latencies to the first active response during the CS-ON and CS-OFF intervals in low players (**C** LP, CS − *N* = 9; CS + *N* = 7) and high players (**D** LP, CS − *N* = 13; CS + *N* = 16), conditioned (CS +) and non-conditioned (CS −). Data are presented as mean + SEM active responses or latencies, binned in 2-min intervals. Significant differences between CS − and CS + subgroups are indicated by *, ** and *** (post hoc pairwise comparisons, *p* < 0.05, *p* < 0.01 and *p* < 0.001, respectively)

Overall analysis of the conditioned suppression data revealed conditioned suppression of alcohol seeking as evident from a significant effect of CS (F_(1,41)CS_ = 11.8, *p* < 0.01), which was dependent on the tone presentation (F_(1,41)tone × CS_ = 11.7, *p* < 0.01) (Fig. 4). Moreover, there was a tone and playgroup dependent difference between conditioned and non-conditioned rats (F_(1,41)tone × CS × playgroup_ = 4.5, *p* < 0.05). Subsequently, the data was further analysed by play group (LP and HP), which revealed significant suppression of alcohol seeking in the HP (Fig. 4B and D), while the LP did not suppress their alcohol seeking upon presentation of the tone (Fig. 4A and C). For the LP, there was no reduction of the number of active lever presses in the CS + subgroup compared to the CS − rats (F_(1,14)tone × CS_ = 0.005, N.S. and F_(1,14)CS_ = 0.58, N.S.) (Fig. 4A). By contrast, the HP showed a reduction in the number of active lever presses upon presentation of the tone in the CS + group compared to the CS − group (F_(1,27)tone × CS_ = 6.8, *p* < 0.05), as well as an overall reduction in responding in the conditioned (CS +) versus the control conditioned (CS −) rats (F_(1,27)CS_ = 11.3, *p* < 0.01) (Fig. 4B). In line with these findings, the HP showed a significantly higher latency to lever press during CS presentation (F_(1,27)tone × CS_ = 6.8, *p* < 0.05 and F_(1,27)CS = 11.3_, *p* < 0.01) (Fig. 4D), while LP did not change their latency to lever press upon presentation of the tone (F_(1,14)tone × CS_ = 0.005, N.S. and F_(1,14)CS_ = 0.575, N.S.) (Fig. 4C).

### Fear conditioning

**Fig. 5:**
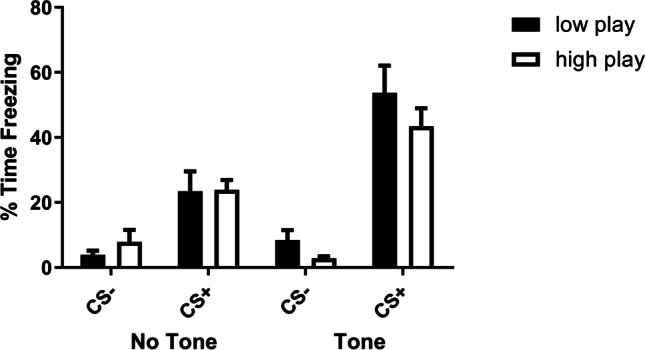
Freezing in low and high playing rats during the 2 min before (no tone) and during 2-min presentation of the footshock-associated CS + (tone) period in the conditioning chamber. The conditioned low and high playing rats (CS +) showed significant context- and CS-induced freezing, when compared to their respective CS − control groups. Group sizes for low playing rats: LP CS − *N* = 9, LP CS + *N* = 7, for high playing rats: HP CS − *N* = 13, HP CS + *N* = 16. The fear conditioning test was performed 1 week after completion of the conditioned suppression test. Data are presented as mean + SEM. ***Significant difference between CS + and CS − groups (post hoc pairwise comparisons, *p* < 0.001)

After the conditioned suppression test, the rats were re-conditioned and the degree of conditioned fear was determined. Analysis of the freezing behaviour of the rats during 2 min before (no tone) and during CS (tone) presentation showed that the CS + rats spent more time freezing compared to the CS − control rats (F_(1,41)CS_ = 94.0, *p* < 0.001) (Fig. 5A and B for LP and HP, respectively). Moreover, conditioned freezing behaviour was augmented upon presentation of the tone (F_(1,41)tone × CS_ = 16.1, *p* < 0.001) and conditioned freezing was comparable for the LP and HP (F_(1,41)tone × CS × playgroup_ = 0.21, N.S.).

## Discussion

Early social development has been proposed to be an important determinant of the risk for addictive behaviours, including AUD (e.g. Innamorati and Maniglio 2015; Stickley et al. 2014; Whelan et al. 2014). Here, we show that individual levels in social play behaviour predict later alcohol consumption, as well as the degree of control over alcohol seeking in rats. That is, rats that displayed high levels of playfulness as juveniles subsequently ingested greater quantities of alcohol, yet showed intact conditioned suppression of alcohol seeking, unlike their less playful counterparts. These findings provide novel evidence for an important role of social play behaviour in the development of the brain mechanisms underlying (resilience to) alcohol use and AUD.

The main finding of this study is that juvenile play behaviour predicts alcohol consumption and the degree of control over alcohol seeking in adulthood. Rats that played most consumed more alcohol. However, the high players suppressed their alcohol seeking in the face of adversity while the low players showed resistance to conditioned suppression of alcohol seeking, indicative of loss of control over alcohol seeking. Conditioned freezing behaviour was comparable for the low and high players, suggesting that the differences in conditioned suppression in this study are not due to differences in fear conditioning, but are rather attributable to differential decision-making with regards to seeking alcohol under adverse circumstances. The rats were trained under a random interval schedule of reinforcement, based on previous studies and with the intention to dissociate alcohol seeking from alcohol-taking behaviour (Limpens et al. 2014a, 2014b; Spoelder et al. 2017; Vanderschuren and Everitt 2004). Because random interval schedules are also known to induce habitual reward seeking (e.g. Dickinson 1985; Nordquist et al. 2007), it would be of interest to determine the relation between play behaviour and habitual alcohol seeking in follow-up studies.

Play behaviour is thought to facilitate the development of social, emotional, cognitive and problem-solving skills (Einon et al. 1978; Graham and Burghardt 2010; Hol et al. 1999; Pellis and Pellis 2009; Piaget 1962; Spinka et al. 2001; van den Berg et al. 1999; Vanderschuren and Trezza 2014; Von Frijtag et al. 2002). For example, Baarendse et al. showed that play-deprived animals display impairments in decision-making and impulse control, particularly under challenging conditions (Baarendse et al. 2013). Further, play deprivation was shown to result in augmented cocaine and alcohol self-administration (Baarendse et al. 2014; Lesscher et al. 2015) and enhanced cocaine-, amphetamine- and alcohol-induced conditioned place preference in adulthood (Walker et al. 2020; Whitaker et al. 2013). Moreover, children who engage(d) more in outdoor play show improved self-regulation and conflict resolution skills (Tremblay et al. 2015). In contrast to previous work on the relation between play and addictive behaviour, the rats for this study were not deprived from social play behaviour. The current observations show that the rats’ tendencies to engage in play behaviour, an individual trait, predict the degree of alcohol use and control over alcohol seeking. These findings complement previous reports and suggest that (more) engagement in play behaviour may contribute to an individual’s flexibility (Einon et al. 1978) and cognitive capacity under challenging circumstances.

Our current findings partly support our hypothesis in that the juvenile animals that played most are indeed less prone to lose control over alcohol seeking. However, the rats that played most consumed more alcohol than the rats with a lower tendency to engage in social play behaviour. Enhanced social play may reflect an endophenotype for a greater overall reward sensitivity. Social play behaviour is rewarding, as shown using different tasks across species (Calcagnetti and Schechter 1992; Humphreys and Einon 1981; Mason et al. 1963; Trezza et al. 2011). Moreover, comparable neural systems modulate social play behaviour and the rewarding effects of substances of abuse (Siviy and Panksepp 2011; Trezza et al. 2010; Vanderschuren et al. 2016). It is therefore conceivable that high playing rats may display a greater sensitivity to rewarding stimuli, as a consequence of which they are more sensitive to engage in play behaviour and to take substances of abuse (Baarendse et al. 2014; Lesscher et al. 2015; Whitaker et al. 2013).

In line with previous reports (e.g. Alaux-Cantin et al. 2013; Amodeo et al. 2017; Milivojevic and Covault 2013; Pandey et al. 2015), we observed an increase in alcohol intake in adulthood after pre-exposure to alcohol during adolescence. However, alcohol pre-exposure did not affect conditioned suppression of alcohol seeking (data not shown). Importantly, adult alcohol intake was determined after an abstinence period of nearly 3 weeks. It is therefore conceivable that the increase in alcohol intake after adolescent alcohol exposure reflects an alcohol deprivation effect, i.e. an increase in alcohol intake after an abstinence phase (e.g. Colombo et al. 2003; McBride et al. 2014; Momeni and Roman 2014; Rodd-Henricks et al. 2000a, 2000b; Serra et al. 2002; Spanagel and Holter 1999, 2000; Vengeliene et al. 2014), rather than an age-dependent risk for AUD. In fact, rats with an adolescent-onset of alcohol consumption were recently shown to retain control over alcohol seeking (Labots et al. 2018).

The differences in the tendency to engage in social play behaviour in rats that were characterized as low and high players were not accompanied by variation in the degree the rats were played with, i.e. the number of occasions they were pounced or pinned upon. This suggests that the differences in play behaviour that we observed reflect variation in the initiative the rats take to play, rather than responding to an invitation to engage in social play behaviour. When comparing the two play sessions, in particular the low and medium playing, rats showed a substantial degree of variation in their tendency to engage in social play across the sessions. Due to this variation in play behaviour between sessions, some animals could not be categorized as a consistent LP or HP and had to be excluded from the analysis. The variation in play behaviour between sessions may be related to the playfulness of the play partner, that could affect the tendency of an individual rat to engage in social play (Pellis and McKenna 1992). Indeed, it is known that play behaviour is affected by the play partner, for instance when combining rats from a low with a high playful strain (Schneider et al. 2016; Siviy et al. 1997) and future studies should address the impact of intra- and cross-group pairings within the Lister hooded strain.

There is a growing appreciation for the need to include both male and female rats in behavioural studies (Shansky and Murphy 2021), so one limitation of the present study was that only male rats were used. There is evidence that male rats tend to be more playful than female rats, although this is somewhat dependent on testing conditions. For example, when rats are observed in mixed-sex groups in home cages, there are robust sex differences with males exhibiting more social play than females (Meaney and Stewart 1981; Meaney et al. 1985). Robust sex differences can also be observed when play is assessed in a neutral testing chamber after a period of social isolation but when rats are tested with littermates with pre-existing social relationships (Pellis and Pellis 1990). On the other hand, when rats are tested for a discrete period (e.g. 5–15 min) in a neutral testing chamber with unfamiliar rats after a period of social isolation, sex differences are less likely to be observed (Himmler et al. 2013, 2014; Siviy 2018). As the rats in the present study were tested with an unfamiliar partner after a brief period of social isolation, robust sex differences in play would not be expected. The extent to which sex and/or gender is a factor in susceptibility to AUD in humans is also complex (McHugh et al. 2018), yet there is evidence that the incidence of AUD in women has been steadily increasing over the last few decades and that women may be particularly vulnerable to some of the negative consequences associated with AUD (Erol and Karpyak 2015; Radke et al. 2021). Having demonstrated a relationship between playfulness, alcohol consumption and control over alcohol seeking in males, it would be insightful to assess females using the current protocol to ascertain whether a comparable relationship also extends to females.

In conclusion, the present study revealed individual differences in social play behaviour in rats that predicted alcohol consumption and the degree of control over alcohol seeking later in life. These findings provide novel evidence for a critical role of social play behaviour in the development of brain and behaviour, in relation to the vulnerability to substance addiction.

## Supplementary Information

Below is the link to the electronic supplementary material.

Supplementary file1 (DOCX 89 KB)
