# Supplementary material for: Individual differences in social play behaviour predict alcohol intake and control over alcohol seeking in rats
Source: Psychopharmacology (Berl). 2021 Aug 2;238(11):3119–30. doi: 10.1007/s00213-021-05929-1 (PMC8605978; doi:10.1007/s00213-021-05929-1)
Supplement: Supplementary file 1 — Supplementary file1 (DOCX 89 KB) [file 213_2021_5929_MOESM1_ESM.docx]

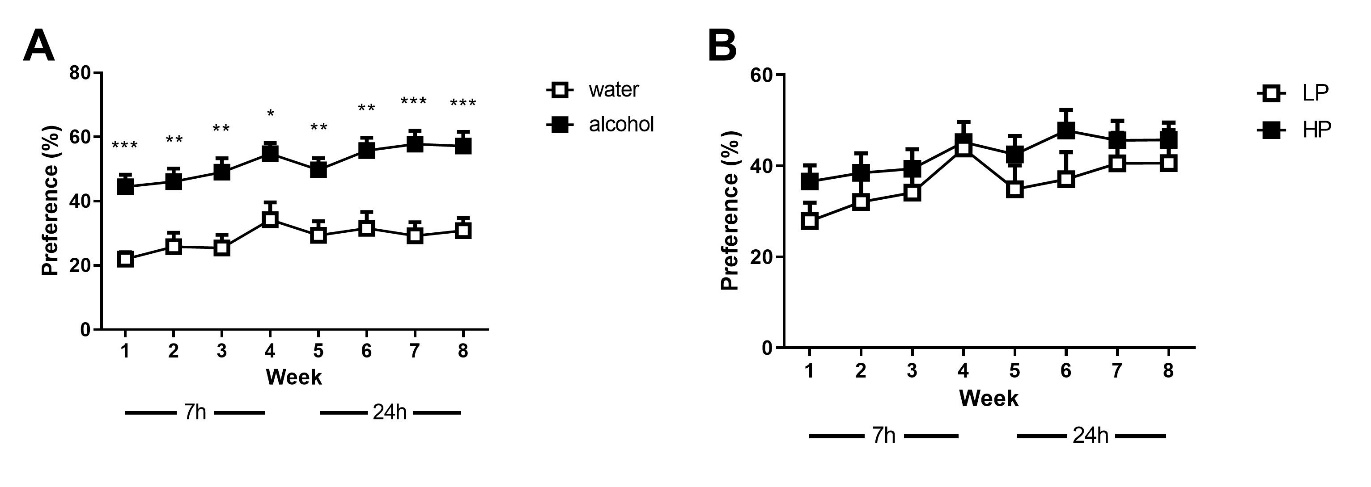


Supplementary Figure 1. Alcohol preference levels for the selected groups of low and high playing rats that were allowed to drink either alcohol or water during adolescence. Panel A shows the preference for alcohol over water in 7h (weeks 1-4) and 24h (weeks 5-8) sessions, by water (N = 22) or alcohol (N = 23) pre-exposure. Panel B shows the preference levels for alcohol for the same rats, grouped by low (LP, N = 16) and high (HP, N = 29) tendency to engage in social play behavior. Shown are average + SEM preference levels per week and by subgroup. Post-hoc pairwise comparisons: *, ** or *** significant difference between adolescent water and adolescent alcohol pre-exposed animals (P < 0.05, P < 0.01 or P < 0.001, respectively).
